# Advancements in 3D Cell Culture Systems for Personalizing Anti-Cancer Therapies

**DOI:** 10.3389/fonc.2021.782766

**Published:** 2021-11-30

**Authors:** Andrew M. K. Law, Laura Rodriguez de la Fuente, Thomas J. Grundy, Guocheng Fang, Fatima Valdes-Mora, David Gallego-Ortega

**Affiliations:** ^1^ Tumour Development Group, The Kinghorn Cancer Centre, Garvan Institute of Medical Research, Darlinghurst, NSW, Australia; ^2^ St. Vincent’s Clinical School, Faculty of Medicine, University of New South Wales Sydney, Randwick, NSW, Australia; ^3^ Cancer Epigenetic Biology and Therapeutics Lab, Children’s Cancer Institute, Randwick, NSW, Australia; ^4^ Life Sciences, Inventia Life Science Pty Ltd, Alexandria, NSW, Australia; ^5^ School of Biomedical Engineering, Faculty of Engineering and IT, University of Technology Sydney, Ultimo, NSW, Australia; ^6^ School of Women’s and Children’s Health, Faculty of Medicine, University of New South Wales Sydney, Randwick, NSW, Australia

**Keywords:** 3D culture systems, personalised medicine, drug resistance prevention, tumor microenvironment, 3D bioprinting, extracellular matrix, microfluidics

## Abstract

Over 90% of potential anti-cancer drug candidates results in translational failures in clinical trials. The main reason for this failure can be attributed to the non-accurate pre-clinical models that are being currently used for drug development and in personalised therapies. To ensure that the assessment of drug efficacy and their mechanism of action have clinical translatability, the complexity of the tumor microenvironment needs to be properly modelled. 3D culture models are emerging as a powerful research tool that recapitulates *in vivo* characteristics. Technological advancements in this field show promising application in improving drug discovery, pre-clinical validation, and precision medicine. In this review, we discuss the significance of the tumor microenvironment and its impact on therapy success, the current developments of 3D culture, and the opportunities that advancements that *in vitro* technologies can provide to improve cancer therapeutics.

## Introduction

Uncontrolled division of neoplastic cells results in the development of a tumour mass composed of a large variety of cellular and non-cellular components, including the heterogeneous population of cancer cells, infiltrating and resident normal cells, extracellular matrix (ECM) proteins and secreted factors. This complex and highly heterogeneous conglomerate of multiple cell types and extracellular components inside of the tumour mass is known as the tumour microenvironment (TME) (1). The interacting networks established in the TME among cancer cells and the other cell types are the key contributors to the hallmarks of cancer and determine the aggressiveness of the tumour (2–4). Furthermore, this tumour heterogeneity within the TME widely contributes to the extent of patient responses to anti-cancer therapies (5). Resembling the network and the heterogeneity involved in every type of cancer is considered one of the most challenging practices among oncology researchers globally. However, understanding the molecular features in the TME of each cancer is fundamental for the successful development of clinically translatable anti-cancer drugs.

## Modelling the Physiology of TME for Drug Testing

The complexity within the TME is propagated by the heterogeneous nature of different tumor entities; that is each individual tumor harbors its own unique intricacies comprised of structural, cellular, genetic, and molecular composition. Our continuous effort to improve our understanding of oncology has led to the development of more effective diagnostic and therapeutic approaches. However, we are also simultaneously unravelling the anomalous disease complexities within cancer that challenges clinical success. In a comprehensive survey of clinical success rates by Hay et al., oncology drugs were found to have only a 6.7% success rate of being approved (6), with other studies estimating as low as 3.4% (7). There are various reasons that contribute to this high rate of failure including 1) inadequate efficacy from poor biodistribution and metabolism of the drug – unsatisfactory therapeutic index; 2) safety concerns associated with significant side effects and off-target toxicities; 3) financial or commercial issues such as insufficient funding or patient recruitment and retention (8–11). Ineffectiveness of therapies is the most common factor (57%) attributed to failure during clinical development (10, 11). Unfortunately, most experimental drugs that were designed through using pre-clinical models to therapeutically target known molecular components are poorly translated to clinical practice.

**Figure 1:**
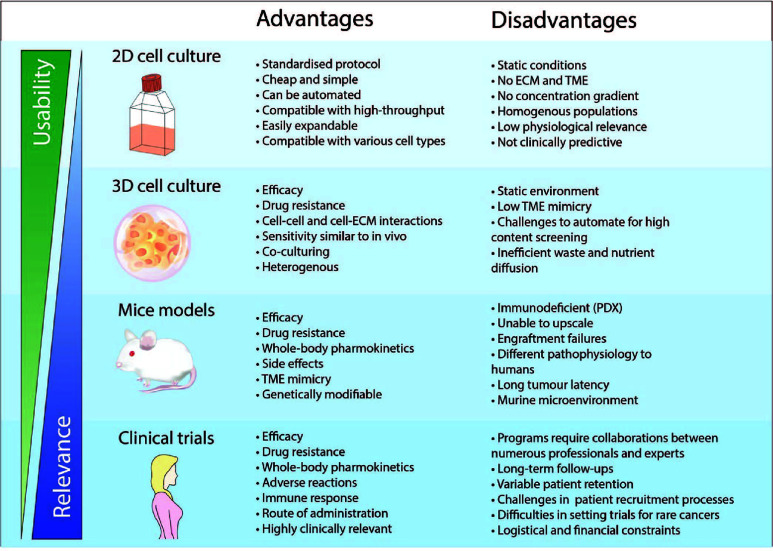
Advantages and disadvantages of drug development using different pre-clinical models and clinical trials. The physical features when using a pre-clinical model is crucial to ensure physiological relevance. 2D cell cultures is a widely adopted and well-established model that has been used consistently in drug discovery and high throughput screening. However, cancer cells cultured in 2D do not recapitulate the biology of an *in vivo* tumor and thus has very poor performance for clinical prediction. As such, the use of more complex models such as 3D cell culture and mice models has been more representative of clinical cases compared to 2D cell culture. However, the standardized implementation of these models for applications in high content screening and personalised medicine remains a challenge.

During the pre-clinical phase, the most commonly employed cancer models are 2D cell cultures before transitioning to *in vivo* mice models (**Figure 1**) (12). Drug testing in animals prior to clinical trials have been a mainstay for determining drug efficacy and toxicity; however, there are also various issues associated with animal models, from increased costs, logistic demand, limited bioavailability, and an increasing ethical concern (13–15). Although these models have provided us with better insights into tumor biology and have made a significant impact on approaches to cancer healthcare, they do not accurately recapitulate the complex TME and molecular features within a human tumor (16, 17). The dismal results of clinical translatability of drugs developed from pre-clinical models highlight the limitations of our current understanding (16). Currently, one of the major obstacles for delivering better cancer patient cares is associated with accurate diagnosis and prediction to therapeutic responses (18). As such, the importance of developing more accurate, cost-effective, and efficient pre-clinical technologies for better *in vitro* and *in vivo* models are crucial to creating more efficacious therapies, predicting therapeutic outcomes, and guiding clinical practice.

### Bridging the Pre-Clinical Gap: 3D Culture Models

**Figure 2:**
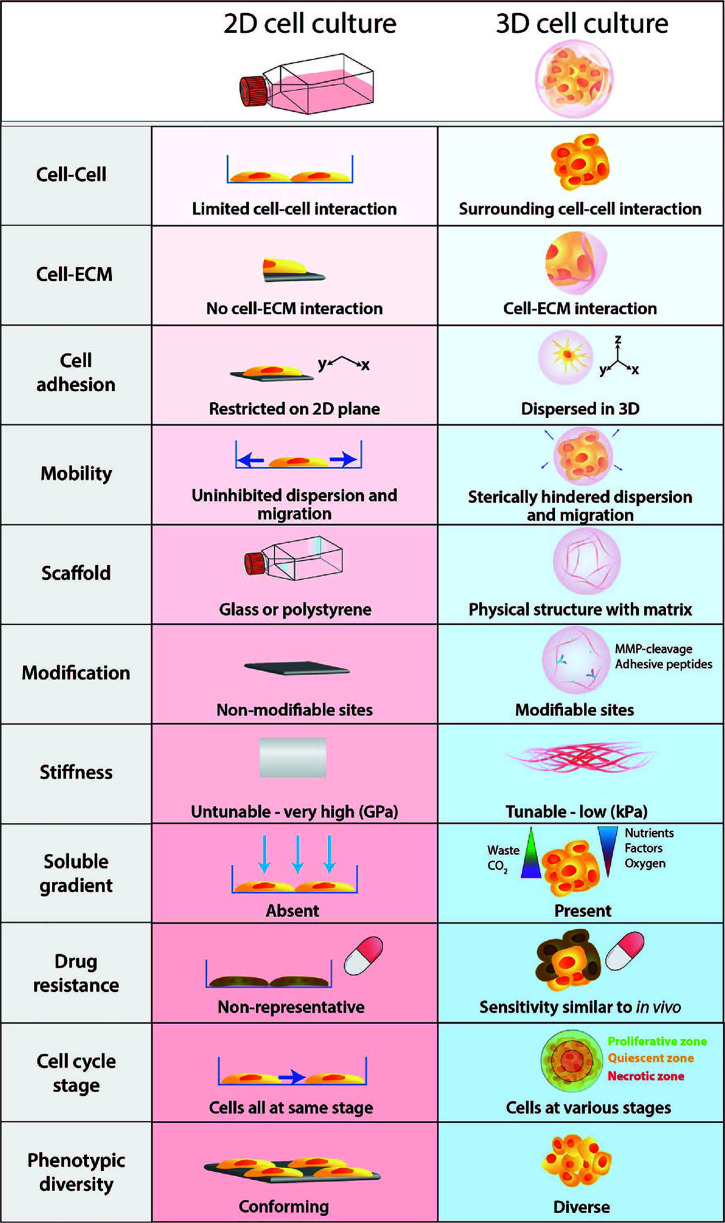
Physiological differences between 2D cell culture and 3D cell culture. Cells develop as a 2D monolayer adopt an apical-basal polarity when plated on a culture flask or a petri dish. The environment that cells are exposed to within the culture flask is a poor representation and does not accurately recapitulate physiological conditions. Comparatively, 3D cultures provide greater biological relevance and cellular response to perturbations are more reflective of *in vivo*.

Many researchers use 2D cell cultures as the *in vitro* pre-clinical model for testing anti-tumor drugs before proceeding with *in vivo* trials (13). This is primarily due to the convenience, simplicity and cost-effectiveness of using a 2D cell culture as a model (**Figure 2**). However, it is evident that results attained from 2D *in vitro* models have almost no clinical translatability to human tumors (13). The 2D monolayer cultures have been optimized to grow on rigid plastic surfaces and thus fail to capture the crucial elements that make up the complex 3D tissue architecture of the TME, which ultimately affects the cellular response of cells to drugs and the off-target effects. While 2D cultures are still predominantly used for drug discovery due to its simplicity and compatibility with high-content screening platforms, 3D culture systems have numerous advantages over 2D cell culture. Thus the transition to 3D preclinical models have become more appealing as improvement in tissue engineering technology has made 3D cell culture more adaptable and tunable over the microenvironmental factors to better reflect the functional pathology of *in vivo* tumors.

The emergence of 3D cell culture models as research tools plays a vital role during early pre-clinical drug development. Recently there has been a paradigm shift in the way researchers study the TME; 3D models are able to better mimic the *in vivo* microenvironment compared to 2D cell culture and their applications are simpler, more efficient, versatile, and cost-effective compared to using animal models (13). Currently, intense efforts are taken to generate new cell lines that represent the vast heterogeneity of tumors. Three-dimensional cultures offer a higher chance to represent the genomic diversity and allow testing of new drugs targeting specific signaling pathways. Additionally, 3D culture is a more efficient way to generate new patient-derived cell lines that fail to grow in 2D. For example, in breast cancer and melanoma, tumor circulating cells derived from patients are successfully grown under hypoxia conditions in suspension cultures (19, 20). And in prostate cancer, organoid models from patient-derived xenografts can be also used to assay drug sensitivity (21).

Furthermore, cells embedded within a 3D matrix self-assemble to form structures more similar to their organisation *in vivo* and enable better intercellular contact and communication. Recent advancement in 3D culture has led to the development of new technologies that can generate more complex 3D cell models that aim to bridge the gap between 2D cell culture and animal models. The improved biological relevance of 3D models is due to several key features: dimensionality, presence of ECM, and concentration gradients (**Figure 2**).

### 3D Cultures – Dimensionality

3D culture models cultivate a more relevant pathophysiological microenvironment that allows cells to aggregate, proliferate, and display phenotypes as they do within the body. The complex cellular interactions between other cells and the 3D matrix are crucial for maintaining regular cell structure, function and mobility. Since cell migration occurs in three dimensions the matrix provides a topology that mimics the 3D architecture of a tissue, allowing cells attach and interact with their surrounding environment (22). The dynamic tensile forces from the matrix play a crucial role in cell migration and are involved in activating pathological mechanisms associated with invasion, ECM remodeling, and metastasis (23, 24). Kock et al. had conducted a study investigating the biomechanical tractions utilized by various carcinoma cells to invade through a collagen gel. Interestingly, the level of matrix contraction was not associated with invasiveness, but rather the cellular adoption of an elongated spindle-like morphology and the complexity of the collagen deformation (24). Furthermore, fibroblasts were reported to migrate more rapidly on a 3D matrix and maintained a more spindle-like characteristic compared to those that were cultured in 2D (25). Contrarily, cells grown on a 2D plane have much less physical hindrance as they move across a planar surface that is only impeded by surface inhibition (23). As such, 3D cultures have been used to elucidate the mechanisms that drive cancer invasion and metastasis. For example, matrix degradation and ECM remodeling are key factors involved in invasive malignancy and have been studied in *ex vivo* models to identify potential targets for cancer therapies, such as inhibiting matrix metalloproteinases and invadopodia formation (26–28).

### 3D Cultures – Extracellular Matrix

The ECM has been well established to influence cell behaviour and response to external factors (29, 30). Cellular phenotypes and functions are dictated by a complex network of signaling that occurs within the context of the microenvironment through cell-cell communications, cell-ECM interactions, soluble factors, and small molecules (29). The importance of these dynamic interactions between cells and its surrounding ECM becomes apparent as cells grown in 3D adopt physical and genetic properties more akin to *in vivo*, such as morphology, phenotype, and expression profiles; whereas 2D monolayers have more vastly different characteristics forced by the unnatural plastic environment (31–33). Additionally, the biomechanical properties of the ECM can modify the signal transductions that occurs within the microenvironment *via* the spatial organisation of cells, stiffness of the matrices, and physical constraints to hinder cell mobility (29, 34). During tumorigenesis, the stiffness of the ECM causes compressive stress that increases the mechanical pressure as the tumor grows and expand. This increased ECM resistance promotes cell-ECM and cell-cell within the tumor communications that can induce hyperactivated mechanotransduction pathways such as RHO/ROCK (35). Consequently, this upregulation of ROCK can increase cancer cell proliferation, migration, epithelial-mesenchymal transition (EMT), and cancer-associated fibroblast (CAF) reprogramming to promote tumor progression (35, 36). Within the ECM various molecules can also regulate the behaviour, differentiation, migration, and phenotypic fates of cells (37). These can include: glycoproteins such as laminin and fibronectin that connects structural molecules together or with cells to orchestrate cell attachment and migration through the ECM; ECM fibres such as collagen and elastin to provide structural elements of tensile strength and elasticity; and proteoglycans such as hyaluronic acid, keratan sulphate, and chondroitin sulphate, that can regulate structural and adhesive properties of the ECM, angiogenesis, and sequester growth factors (37, 38). Additionally, drug sensitivity in cells can be variable based on cell-ECM interactions and spatial positioning of cells relative to the ECM (30, 39, 40). Changes in ECM composition and its biophysical properties do not only alter cell phenotype but can also regulate the cellular response to drugs, such as promoting acquired resistance or reducing drug accumulation within the tumor (41, 42).

### 3D Cultures – Concentration Gradient

Soluble metabolites, oxygen concentration, and pH throughout the TME can strongly affect the tumor pathophysiology and the efficacy of therapies (39–41). These components exist as a gradient within the tumor; peripheral cells in closer proximity to blood vessels have more access to soluble constituents and oxygen, which decreases as it diffuses through the ECM to the tumor core. The concentration gradients of growth factors, nutrients, wastes, and gases compounds to the intratumoral heterogeneity and influences the signaling within the microenvironment including cell function, proliferation, morphogenesis, and chemotaxis (30). As such, cells grown in larger 3D aggregates also mimic the *in vivo* condition by existing in various proliferative states based on nutritional access that is restricted by the concentration gradient. From the peripheral to the core of the spheroid is composed of the outer proliferative zone, semi-peripheral quiescent zone, and the central necrotic zone, where each region is in different cell cycle stages (34). This difference in cell cycle stage amongst cancer cells in 3D cultures also contributes to the variable sensitivity of drugs and tumor recurrence from quiescent cells (32, 34). Since blood vessels are unevenly distributed throughout the tumor, regions with low or absent vasculature are hypoxic and acidic and contain high interstitial oncotic pressure (43). In the context of pharmacokinetic, the concentration gradient limits the penetrance of drugs through the tumor and attains a dosage sufficient to exert their therapeutic effects on all the cancer cells (44). In addition, the half-life of drugs also determine the distribution of the agent throughout the tumor; drugs with a long half-life will have more uniform distribution across the tumor even if the rate of the diffusion is low, whereas drugs with a short half-life will have a nonuniform distribution (45). Most research also focuses on the role of mechanisms of action for drugs or therapy resistance, however the physiochemistry of drugs is often neglected (44). As a result, the impeded distribution and diffusion of pharmaceutical agents through the tumor still remains one of the major challenges in anti-cancer treatments. This important, yet often overlooked, the property makes 3D cultures a more accurate model to study the impact of pharmacokinetics and even bacterial biodiversity (46) from concentration gradients (47); compared to cells in 2D cultures which are all homogenously exposed to nutrients and agents (30).

### 3D Cultures – Microbiome

The clinical research on the association of microbiota and cancer started in 1868 by William Busch. After centuries of research, increasing evidence implicates that microbiota influences the TME, tumor metabolism, and tumor immunotherapy response (48). For instance, gut microbiota dysbiosis may induce breast tumorigenesis (49). The influence of microbiota in tumourigenesis and tumor progression may differentially impact different types of tumors, as it has been demonstrated the existence of tumour type-specific intracellular bacteria (50). This tumour microbiome diversity, specificity and relevancy provide both challenges and possibilities for tumour treatment (51). Modelling the interactions of microbiota and tumour offers an efficient method to understand the inner correlations and evaluate the microbiota-target drugs.

Compared to the 2D cell models, the 3D culture can replicate the mechanical cues of solid tumors and the chemical gradience (pH, hypoxia, lactate, etc.), which influence the microbiota proliferation, distribution, movement, variety, and metabolism. This, in turn, could affect the metabolite levels in the TME, for instance, by regulating the gene expression (52). Stem-cell derived organoids, relying on 3D culture, have become indispensable tools to investigate the host-microbiota interactions (53). For instance, intestinal organoids usually form luminal structures within the hydrogel’s matrix where the bacteria of interest can be microinjected (54). As such, stomach organoids were modelled with Helicobacter Pylori (55). The organ-on-chip approach could also mimic the complexity of 3D tissues or tumors, which attracts more attention to the study of microbiome and disease, an example of this approach has been applied to the gut-microbiome on a chip (56, 57). The bidirectional interactions of drugs with local microbiota manipulate the host response to chemotherapeutic drugs (49, 51, 58, 59), which potentially highlights the importance of 3D cultured models in pharmomicrobiomics. In addition, with the fast development of engineered microbial therapies, 3D cultures become a good candidate for more reliable screening, enabling parallel and long-term monitoring (60).

## Approaches to 3D Culture Models

Anti-cancer drug screening and the development of new personalised therapies are primarily conducted in 2D cultures of cancer cell lines (30). Researchers have generated the Cancer Cell Line Encyclopedia to help provide predictive modelling of anticancer drug sensitivity (61, 62). 2D cultures are a mainstay in biological research and have provided us with a deeper insight and understanding of cancer mechanisms, biomarker discovery, and stratification of tumour profiles. From a drug-development perspective, the improvement of more predictive preclinical models is essential to permit the earlier dismissal of drug candidates from clinical trials and reduce pharmaceutical cost – the development of a new drug is estimated to be $2.6billion (63). The disparate response to therapies observed in 2D cultures and in mouse models becomes evident in clinical trials, in which oncology drugs are known to have as low as 3.4% success rate (7). For example, the drug Palifosfamide was a DNA alkylating agent used as a first-line treatment for metastatic soft tissue sarcoma that had failed in Phase III PICASSO 3 trial due to not being able to meet its primary endpoint of progression-free survival in patients (NCT01168791). Within the lab, Palifosfamide demonstrated cytotoxicity in sarcoma cell lines with an IC_50_ range of 0.5-1.5ug/mL and treatment in xenograft SCID mice resulted in tumour growth inhibition and improved event-free survival (64).

**Figure 3:**
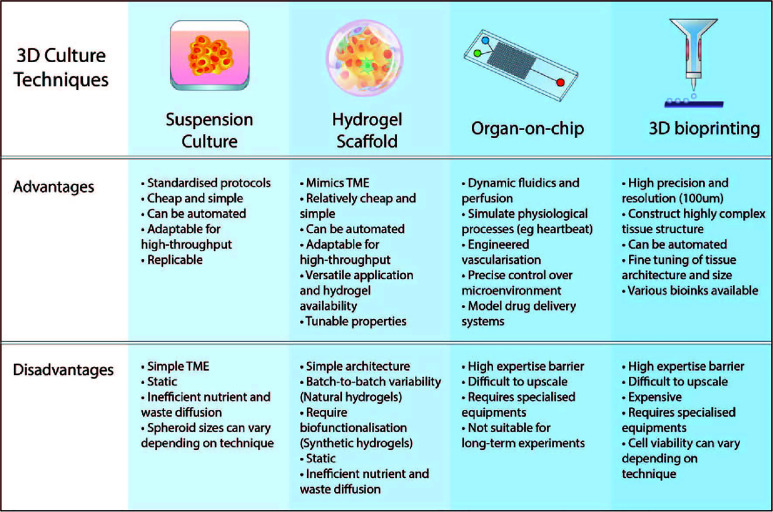
Advantages and disadvantages of various 3D culture approaches. The key features of 3D culturing aim to improve the biomimicry and predictive value of pre-clinical models. Suspension cultures and scaffold-based approaches are easier to implement in the lab and upscale for high-throughput. Advancements in microfabrication technology such as microfluidic chips and 3D bioprinting have resulted in more complex and physiologically-relevant models that can be generated.

Recapitulation of the fundamental tissue environment within the human body is essential for the proper evaluation of drug effectiveness. From both the cellular populations to the acellular compositions, such as the ECM, pre-clinical models aim to replicate both pathophysiological and healthy bodily functions. Mimicking the complexities of all the biological processes in a single model is highly challenging. Therefore, researchers are developing new techniques to make 3D culture more applicable and easier to implement (**Figure 3**). As such, 3D cell cultures are becoming more convenient and accessible while allowing researchers to improve upon the traditional *in vitro* 2D cultures, aiming to model more native-like interactions of tissues to study their mechanisms.

### Suspension Cultures

Spheroids are grown as aggregates in suspension and have been applied in various cell types, such as cancer cells, hepatocytes, and stem cells (65). Additionally, they can be grown as a monoculture or together as a co-culture with other cell types to provide more physiologically relevant interactions. In a study by Courau et al., colon cancer cells were co-cultured with T cells and NK cells to evaluate tumour-lymphocyte communication and test immunomodulatory antibodies (66). Spheroids are able to recapitulate the *in vivo* characteristics of intercellular communications, cell-ECM interaction, and behaviour. The size of spheroids are dictated by the initial seeding cell number; thus it is crucial to optimize the culture conditions to ensure that the spheroids do not become too large and suffer from hypoxia and necrosis from poor nutrient diffusion (30). Spheroids can be generated through 1) hanging drop; 2); low adhesion plates 3) magnetic levitation.

The hanging drop technique is one of the earliest methods of developing 3D cell culture (67). This technique uses specialized hanging drop plates that contain a bottomless well where the droplet of media forms. Cells aggregate within the small droplet of culture media to generate the spheroid over several days. Co-culturing can be conducted by adding cells during the initial dispensing or from consecutive addition of the cells (65). However, transfer of the spheroids from the hanging drop plate to another non-attachment plate will be necessary if growing larger spheroids or downstream assays. The hanging drop technique is relatively facile and efficient and has been adapted for use in various cell lines for toxicity testing and drug screening (68, 69). This technique has very high reproducibility with consistent size spheroids (70).

Low adhesion plates have a low attachment coating on the surface of the wells that reduces cell adherence and promotes cell aggregation into spheroids. The coating can include the non-adherent poly-HEMA or agarose (30). Larger volumes of media can also be used in the low adhesion plate allowing a more efficient generation of tumour spheroids. Furthermore, low adhesion plates are designed for high-throughput screening, allowing 3D cell culturing and assaying within the same, unlike the hanging drop technique (65).

Magnetic levitation generates spheroids through the use of magnetic nanoparticles. Cells are incubated with the nanoparticles for several hours to overnight and are then loaded in a low adhesion plate. The low adhesion plate minimizes cell adhesion to the plate while the application of a magnetic field above the plate incites cells to aggregate and produce the spheroids, which can be maintained without requiring a continuous magnetic force. The spheroids can then be subsequently manipulated using other magnetic tools, such as to accelerate cell migration (71). Magnetic levitation can be scalable for use in high throughput screening and drug discovery (72).

### Hydrogel Scaffold Models

Biomimetic scaffolds that model the ECM have been developed over the past few decades to develop microenvironments that can overcome the limitations of traditional 2D cell cultures. In particular, hydrogels have gained interest as physical support that provides the architecture, topology, and biomechanical properties which enables more *in vivo*-like cellular behaviour and communication. Hydrogels can be used to generate various natural and synthetic ECMs that simulate the microenvironment and stiffness of most soft tissues (29). The internal structures of hydrogels consist of networks of cross-linked polymers that can be moulded through mild gelation conditions that have minimal cytotoxicity (13). Furthermore, hydrogels can be chemically modified to tailor matrix stiffness and viscoelasticity (73–75). Integrin interaction (76, 77), growth factor binding (78), and the 3D organisation of the cells (79) can be tuned through the decoration of hydrogel with a variety of peptides (80, 81). ECM remodeling and cell migration can be facilitated through the inclusion of degradable MMP cleavage sites (76, 77), while the synthetic ECM environment can be enriched with matrix proteins including collagens (82), laminins (83), and fibronectin (84), as well as critical matrix molecules such as hyaluronic acid (hyaluronan) (85). This customizability allows hydrogels to have extensive application and versatility in biological research by offering a range of physical and biochemical characteristics.

Natural hydrogels are derived from sources that are inherently biocompatible (29). Various ECM constituents have been derived from materials such as collagen, fibrin, hyaluronic acid, alginate, and the commercial product Matrigel, a reconstituted basement membrane extracted from murine sarcoma cells (86). These hydrogels have various endogenous factors that promote bioactivity and sustain natural cell function, proliferation, and differentiation. For example, collagen is a widely used ECM that orchestrate controlled cell migration, proliferation, and response to therapies through alteration in stiffness and collagen concentration (87, 88). A study from Puls et al. had studied the progression of metastasis in pancreatic cancer using 3D matrices created with type I collagen and found that exposure to fibrillar collagen induced EMT (89). Increased density of collagen fibril resulted in closer arrangements of cell clusters and matrix stiffness (89). Alginate is also a natural polymer derived from brown algae that can gelate *via* ionic crosslinking of the polysaccharide backbone by divalent cations, such as calcium, magnesium, or barium (73, 90). The stiffness of alginate hydrogel can be modified based on the level of cross-linking that is dictated by the concentration of the crosslinking agent. Importantly, alginate gels are inert as they do not contain any mammalian cell adhesion ligands, and with their low protein adsorption, makes them ideal as a matrix for the encapsulation of cells and tissue (91). Additionally, alginate gels under neutral pH and room temperature, resulting in minimal cellular disruption under gelation conditions (90). Alginate can be biofunctionalized with the addition of adhesive and hydrolytic moieties and has been used as a matrix for various biomedical applications (92–94). A key advantage of alginate matrices is that cells can be easily recovered by dissolving the alginate with a chelating agent, such as sodium citrate. Recently, alginate matrices have been proposed for drug screening in breast cancer tumoroids derived from tumour pieces that retain luminal mechanics (95). Hyaluronic acid is another natural hydrogel that has major biomedical applications due to its high moisture retention and viscoelasticity (96). Hyaluronic acid is a non-immunogenic polysaccharide that is found ubiquitously in the ECM in epithelial and connective tissues and is involved in wound healing, inflammation, and embryonic development (96). It can be modified with functional groups allowing for a diverse range of applications in regenerative medicine, oncology, and bioengineering (97–101). However, some drawbacks of natural hydrogels can include poor control over the gelation condition, uncontrolled polymer network structures, lower mechanical integrity, and lower experimental reproducibility due to batch-to-batch variations (65, 86).

Synthetic hydrogels are inert scaffolds that permit a higher degree of modification for desired biological or physical conditions, such as biodegradability, porosity, functionalization with adhesive peptide sequences, growth factors or cleavage sites (29, 30). Compared to natural hydrogels, synthetic gels are cheaper and add improved experimental reproducibility as it has a lower batch to batch variation during manufacturing and can be adapted to suit the research need. However, the disadvantage of most synthetic hydrogels is that they act as a minimalistic matrix and have a less complex microenvironment due to the lack of endogenous factors that are generally present in natural hydrogels (29). As such adhesive moieties and catalytic sites need to be crosslinked into the synthetic scaffold to improve their biofunctionality, such as peptides that can mimic fibronectin or laminin-integrin binding (102, 103). Various non-natural sources can be derived to produce these matrices, such as polyethylene glycol (PEG) (104, 105), polyvinyl alcohol, and polylactic acid (PA) (30, 106). PEG has been used for various 3D culturing and tissue engineering applications. For example, PEG has been cultured with breast cancer cells and CAFs to evaluate drug resistance through pathways associated with tumour-stromal interactions (107, 108). In another study, Caiazzo et al. found that PEG can facilitate pluripotency by manipulating the microenvironment of the matrix to create a “reprogramming niche” that promotes MET and increased epigenetic remodeling capable of shifting the somatic cell fate (109). Biomechanical strain and tension induced by the matrix have been reported to modulate the epigenetic and transcriptomic state of cells as a response to their surrounding environment (110–112).

### Microfluidics System

The advancement in microfabrication technology has led to the development of microfluidics systems that provides more dynamic microenvironments. These systems are designed with specific structures and scaffolds that can be manufactured through patterning techniques such as soft lithography, photolithography, and micro-contact printing (65). Microfluidics permits precise control over small volumes of fluid through hollow channels that can be smaller than 1µm in diameter (13). These devices or chips have been an essential development in microsystems technology that can generate and manipulate the fluid flow and spatiotemporal gradients to improve the biological relevance of *in vitro* models (113). Nutrients, drugs, and wastes can be readily delivered or removed *via* continuous perfusion through the microchannel (114). Within the microfluidic system, spheroids can be generated at high throughput and with a precision that are uniform in size for both monocultures and co-cultures (115–117).

Microfluidic technology has been used to create more cost-effective and accurate biomedical models to test the pharmacokinetics, efficacy, and toxicity of treatments. The internal dimensions of a microfluidic chip can be composed of multiple channels – depending on the design and application – where the size of structures can be between the micrometer to millimeter range (118). Generally, microfluidic chips are manufactured using an inert and non-toxic polymer as a base material, such as poly-dimethylsiloxane (PDMS) (23). The microfluidics control and miniaturization of the whole system present several key benefits: 1) high-throughput capabilities; 2) cost-efficient and low consumption of reagents – within the nanoliter to picolitre range; 3) fine-tuning of conditions and automation (118, 119).

A major approach of the microfluidic system is developing organ-on-chip which is able to create a complex *in vitro* model that recapitulates more organ-specific microenvironments. Organ-on-chip focuses on capturing the critical aspects of the normal biological functions or disease states of the organ of interest. This allows researchers to investigate disease phenotypes and pharmacological responses that are clinically relevant and provide more accurate predictions of treatment efficacy (65, 120). Nutrients, growth factors, oxygen, and drugs can be circulated through the chip as a continuous supply *via* dynamic perfusion which can be automated – in addition to waste removal (12). The controlled fluidic motions can also be used to mimic various mechanical signals including shear stress; compressive forces; physiological flow, such as blood flow; and tissue-specific motions, such as cardiac rhythms and respiratory (120–122). Consequently, microfluidics chips have been used to recapitulate aspects of the TME for anti-cancer drug developments, circulating cancer cell detection in blood samples, and personalised organ-on-chips (123–126). The simplest tumour-on-chip models have been applying 3D spheroids within a microfluidics system (127–129). However, more sophisticated tumour-on-chips platforms have been developed that utilizes the dynamic flow of microfluidics. In a study by Chen et al, an *in vitro* breast tumour model was created on a chip to evaluate nanoparticle-based drug delivery systems (130). This chip included a layer of endothelium that lined a microvessel wall, the ECM and tumour spheroids to generate a real-time drug delivery model. Treatments such as doxorubicin – a standard of care therapy for breast cancer – was loaded in carbon dots to study the penetrance of the treatment through the endothelium to the spheroids, where the efficacy and cytotoxicity of the drug delivery were assessed using *in situ* assays within the same system (130). Tumour-on-chips can also contain engineered vascularization as part of the model using perfusable system to imitate the flow of blood vessels to more closely mimic other mechanisms within the TME, including metastasis, angiogenesis, and drug metabolism (131–134). Argwal et al. discovered that vascularized *in vitro* 3D breast tumors exhibited significantly higher resistance to doxorubicin compared to avascular 3D tumors (4.7 times) and 2D culture cells (139.5 times) (135). Interestingly, this high drug resistance could also be overcome *via* a nanoparticle-based drug delivery method (135). The inclusion of vascularization and dynamic flow has also allowed researchers to study the pathophysiology of blood-based cancer with *in vitro* models, such as lymphoma (136).

### 3D Bioprinting

The development of *in vitro* 3D models that increase the probability of preclinical drug research representing patient outcomes in drug trials, and potentially remove the need for animal studies, may render preclinical cancer research more cost-effective and accessible. However, the use of novel 3D models in cancer research remains restricted by model reproducibility; a prerequisite for specialized training and limitations relating to throughput. The development and commercialization of 3D bioprinting technologies offer an exciting solution to these challenges. 3D bioprinting is an additive manufacturing process defined by the creation of a 3D structure through controlled and typically automated deposition of a biocompatible material or ‘bioink’. This advanced technology is capable of accurately constructing complex tissue structures that faithfully recapitulate native *in vivo* architecture (137). 3D structures can be created directly from highly viscous or shear-thinning bioinks, where the bioinks can be mixed with the cell suspension to generate functionalized cell models. Alternatively, printed bioinks that are less viscous can be solidified through the addition of other chemicals, cooling, or exposure to light or heat (138).

Bioinks are printable, biocompatible solutions that comprise the necessary elements of a desired 3D microenvironment. Bioinks vary greatly in their composition depending on the printing method and the application. Cells, native proteins, growth factors, and signaling molecules can be combined with synthetic compounds that are both printable and biomimetic. Synthetic molecules can likewise be decorated with peptide sequence (139), MMP degradable ligands and drug molecules (140) so that they are more biocompatible, biodegradable or bioactive. Modifications to the bioink properties and bioprinting methods can be tuned to tailor to the desired applications and studies. For example concentrated bioinks may be necessary for creating dense, stiff structures such as bone biomimetics (141), or dense tumour microenvironment models (142). However, concentrated bioinks are highly viscous and result in increased cell death during printing due to high shear forces. As such, it is also important to optimize these modifications to ensure compatibility with the cell types.

Most 3D bioprinting strategies involve droplet, extrusion, and stereolithographic-based structure creation – for an extensive review on the methods refer to the reviews (138, 143). Commonly employed 3D bioprinting processes include 1) droplet-based 3D bioprinting (DBB), which uses sequential depositions of discrete bioink droplets to create structures (144); 2) drop-on-demand bioprinting (DOD), a subcategory of DBB that controls droplet size and placement by regulating the position and ejection of bioink from a nozzle (145, 146); and 3) laser-assisted bioprinting, an alternative DBB technology that propels bioink droplets from an inverted ‘donor slide’ onto a receiving slide using localized heating of a substrate sensitive to laser radiation (147, 148).

Each 3D bioprinting strategy has various, often interlinked, tradeoffs and downstream applications. For instance, extrusion-based 3D bioprinters create structures by layering continuous beads of bioink from nozzles, whereas stereolithographic 3D bioprinting uses light to cure regions of bioink precursor within a bath, building a structure layer by layer (143). In this case, stereolithography limits printing to a single bioink at a time but is excellent for creating complex networked microarchitecture. For example, this has been used to create osteoblast and MSC-laden bone biomimetics (141) and replica microvasculature (149), which were seeded with invasive cancer cells to simulate metastasis and investigate cancer cell migration. Furthermore, the placement of ink on a printing surface is less complex in extrusion printing compared to droplet-based systems where droplet size, flight and placement vary with ink properties (150). However, printing with droplets offers an advantage in throughput and high-resolution patterning as the same nozzle set of a DOD system simultaneously creates multiple structures comprising many different bioinks. Extrusion printing has been used in the creation of large 3D structures to investigate glioblastoma-macrophage interactions (151), and meshes of cervical cancer (152), lung adenocarcinoma (153) and mammary epithelial cells (142) for 3D cancer modelling and drug screens. Conversely, the throughput advantage offered by DOD bioprinting has been exploited to create arrays of hepatic and brain cancer cell lines for drug screening (154), and co-culture patterning of ovarian cancer cells and fibroblasts for investigations of cell interactions and paracrine signaling (155).

There is currently a matter of contention in 3D bioprinting created by the conflicting practices of requiring printing processes to be completed quickly, and simultaneously allowing complex 3D models sufficient time to develop and mature. Bioprinting exposes cancer cells to reagents, processes and forces that fall outside their typical environmental niche. As such, reducing the time for which cells are exposed to the reagents and forces improves cell viability and preserves the *in vivo* biology critical to accurate tumour model creation (138). However, the biological processes central to the development of histological micro-architecture are rarely static, proceed slowly and require time to develop. There is a tendency within 3D bioprinting to emphasize time reduction and to prioritize the rapid completion of printing procedures (143). Yet incorporation of time-related factors and processes will be critical as our general understanding and mastery of 3D bioprinting progresses and becomes further integrated into cancer research.

The term ‘4D bioprinting’ has been used to describe 3D bioprinting strategies that integrate the changing of printed structures over time (156). These strategies may rely on organically occurring biological processes such as matrix deposition, tissue self-organisation and cell differentiation (25). Brassard et al. relied on biological dynamics to create complex macro-structures reminiscent of vascular, connective and gastrointestinal tissues (157). These structures were self-assembled from concentrated cell solutions printed into an ECM hydrogel prior to gelation. The creation of *in vitro* organoids is critical for translatable studies into cancer cell behaviour and drug toxicity. Similar concepts are also being embraced to replicate and investigate the tumour microenvironment directly. For example, Yi et al. created an advanced glioblastoma brain cancer model with initial depositions of silicone ink, endothelial and tumour cells (158). Maturation of the model led to the formation of various features typical of glioblastoma including necrotic foci and pseudopalisades within the tumour cell mass, and leaky endothelial microvessels (159, 160).

In addition to internal biological drivers, externally controlled stimuli can be used to modulate cell behaviour and the printed material surrounding them. The creation of dynamic 3D printed structures is critical for studying the ECM remodeling integral to tumour growth, cell metastasis and drug permeability. Studies have used various stimuli including temperature (161), pH, osmolarity (162), light (163, 164), humidity, magnetic force (165) and electrical charge, to affect material stiffness, size, density, binding affinity (166) and molecular organization (166) of responsive ‘smart’ materials. Stimuli may cause unidirectional irreversible material responses, or they may be bidirectional and reversible (161). Responses can also be stacked, allowing multiple different material states. In an example of this, Tabriz et al. enabled a multistage crosslinking of printed alginate structures through the addition of sequential Ca^2+^ and Ba^2+^ solutions (167). Each stage further increased the printed structures’ durability, facilitating both the initial printability of the bioink, as well as its long-term stability under culture conditions. Aside from material properties, external stimuli can be used to alter the shape of printed structures. Gladman et al., used anisotropic swelling to create complex dimensionality, folds and curvature in 3D planar printed shapes (168). A similar concept was used in a ductal carcinoma study to create geometric mimicry of mammary ducts and acini (169). The impact of responsive bioinks on cancer research is yet to be fully realized. However burgeoning developments in stimulus-responsive geometry and embracing temporal biochemical and biophysical dynamics offer the potential for 3D bioprinted models to be shaped by factors outside of printing complexity (170).

The ability to create representative *in vitro* models is progressing and our understanding of 3D cellular biology continues to grow. To leverage the advances made in these areas within cancer research, the throughput and reproducibility made possible through 3D bioprinting will be critical. Economically viable cancer research requires *in vitro* models that are not only representative of physiological and pathological conditions, but that can be created quickly and efficiently. For this to be possible, we require 3D advanced bioprinting techniques that exploit both intrinsic cell behaviors and innovative biomaterial developments. Synthesis within these areas offers interesting future opportunities for complex 3D model development and the attainment of critical cancer research goals.

## Limitations of Technology in 3D Models

Although 3D culture has been demonstrated to show great promise as a pre-clinical model, a major drawback of 3D cultures is in their implementation for high-throughput screening; a vital aspect for high-content screening and drug development (171, 172). In particular, three significant technical challenges hamper the adoption of 3D culture technology for high-throughput screening: 1) the automation of liquid handling in 3D culture; 2) culture optimization and assay variability; and 3) automated imaging and visualization of 3D structures. The automation of liquid handling can be conducted in suspension cultures such as through the use of ultra-low-attachment microplates or hanging drop technique (30). However, the application of automated liquid handling translates poorly when using hydrogel-based techniques, such as Matrigel. This is primarily due to the undefined compositions between batches that impact reproducibility and consistency and require highly controlled working environments and rapid processing due to their temperature-sensitive gelation conditions (173). Additionally, this batch-to-batch variation in natural hydrogels considerably impacts cell culture conditions and assay quality and reproducibility; as such it is crucial to ensure consistency between batches when conducting high-throughput screening, such as ECM composition and protein content (103). Finally, 3D models permit co-culturing of multiple cell types and provide a higher morphological complexity compared to 2D cultures; allowing improved multiparametric analysis of cell response to drugs. The additional parameters are particularly valuable as they provide a more accurate evaluation of the efficacy and mechanisms of pharmaceutical agents (174). However, this dimensionality also poses a difficulty in computational image analysis and visualization. The complex topology and thickness of 3D models make them it incompatible with most automated imaging systems due to low light penetration and absorption across the multi-layered structures (103). As a result, this can introduce an imaging bias in which only the exterior cells – the layer where cells are exposed to the highest concentration gradient for nutrients and drugs – are imaged and the internal cells are excluded. Despite these challenges, new culture platforms and imaging systems are being developed that aim to overcome these technical difficulties to create 3D cultures that are amenable for high-throughput screening. These developments include using synthetic hydrogels to generate more consistent 3D cell cultures; automated high-resolution imaging using light-sheet microscopy; and integrated computational platforms for data analysis and visualization of 3D cultures (175–177).

## Conclusions

The improvement in 3D culture technology has led to the generation of *in vitro* models that can encompass more physiological and tissue-specific microenvironments with the aim to overcome the drawbacks observed in other pre-clinical models and have better predictive value for clinical outcomes. 3D culture models allow researchers to recreate specific pathophysiological conditions and tumorigenic processes to identify potential biomarkers for therapeutic targeting or assessing cell response to therapies and drug efficacy. Currently, there has been significant interest in using primary clinical samples in 3D culture for personalised drug screening platforms to improve clinical outcomes and reduce side effects (178, 179). Although there are still practical challenges in the widespread adoption of 3D cultures, advancements in this field will provide researchers with a powerful tool to dissect disease mechanisms, identify new biomarkers, provide valuable data in drug development, and realize the potential in the next generation of personalised medicine.

## Author Contributions

Writing— original draft preparation, review and editing by AL, LR, TG, GF, FV-M, and DG-O. Figures designed by AL. Supervision by FV-M and DG-O. All authors contributed to the article and approved the submitted version.

## Funding

AL is supported by a UPA Scholarship from UNSW. LR is supported by a UIPA Scholarship from UNSW Sydney and a PhD top-up award from Kids Cancer Alliance (KCA). FV-M holds a Cancer Institute New South Wales Fellowship (CDF181218). DG-O is holds the Elaine Henry Fellowship from the National Breast Cancer Foundation (NBCF) of Australia (IIRS-21-096) and is supported by a Cancer Council New South Wales (CCNSW) grant (RG18-03).

## Conflict of Interest

TG is employed by Inventia Life Science Pty Ltd as stated in affiliations.

The remaining authors declare that the research was conducted in the absence of any commercial or financial relationships that could be construed as a potential conflict of interest.

## Publisher’s Note

All claims expressed in this article are solely those of the authors and do not necessarily represent those of their affiliated organizations, or those of the publisher, the editors and the reviewers. Any product that may be evaluated in this article, or claim that may be made by its manufacturer, is not guaranteed or endorsed by the publisher.
